# The Measurement Performance of the Parkinson's Disease Activities of Daily Living, Interference, and Dependence Instrument

**DOI:** 10.3389/fneur.2022.760174

**Published:** 2022-03-31

**Authors:** Linda S. Deal, David A. Andrae, Daniela E. Myers, Nathan Johnson, Brandon Foster, Christopher J. Evans

**Affiliations:** ^1^Patient-Centered Outcomes Assessment, Pfizer Inc. New York, NY, United States; ^2^Outcomes Research, Endpoint Outcomes, Boston, MA, United States; ^3^Health Economics and Outcomes Research, Pfizer Inc. Collegeville, PA, United States; ^4^Outcomes Research, Endpoint Outcomes, Long Beach, CA, United States

**Keywords:** Parkinson's disease, patient-reported outcomes, activities of daily living, reliability, validity

## Abstract

Parkinson's disease is a neurodegenerative disease that can be associated with motor fluctuations that result in substantial negative impact to an individual's activities of daily living. Understanding the patient's perspective about the impact of Parkinson's disease therapies is an important part of drug development and shared treatment decision-making. The objective of this research was to examine the structure, scoring, internal consistency, test-retest reliability, and concurrent and known groups validity of the Parkinson's Disease Activities of Daily Living, Interference and Dependence (PD-AID) instrument, a new, patient-reported outcomes instrument, developed to assess the clinical benefit of Parkinson's disease treatment from the patient's perspective. This was a non-interventional study among persons with mild-to-moderate Parkinson's disease currently using and responding to L-Dopa. The structure of the measure was confirmed applying item response theory to data from baseline, supporting 4 candidate scores. Baseline Patient Global Impression of Severity ratings facilitated known-groups analysis. Data from all participants were used to estimate test-retest reliability. Concurrent validity was assessed using correlations with related measures. Participants (*n* = 94) were mean age 69 years (mean time since diagnosis 6.9 years); 34 experienced L-Dopa-related dyskinesia. Psychometric models supported 4 candidate scoring regimes for the PD-AID. All exhibited adequate reliability and validity characteristics and strong internal consistency. Correlations with reference measures were in the expected direction and range of magnitude. Analyses supported the PD-AID as fit-for-purpose, producing psychometrically sound scores. Further research to confirm the measurement properties of the PD-AID in an expanded sample and to establish thresholds for meaningful score changes is recommended.

## Introduction

Parkinson's disease (PD) is a progressive neurodegenerative disease affecting more than 10 million people worldwide (1). The cardinal signs include tremor, rigidity, bradykinesia, and impaired balance (2). PD is frequently associated with motor symptoms caused by the loss of dopamine neurons in the basal ganglia and the substantia nigra. Levodopa (L-Dopa), an exogenous source of a dopamine precursor, remains the most effective and widely used pharmacotherapy for PD; however, its prolonged use is characterized by fluctuations in efficacy and disabling dyskinesias that negate its beneficial effects and are difficult to treat (3).

The Parkinson's Disease Activities of Daily Living, Interference, and Dependence (PD-AID) is a new patient-reported outcome (PRO) instrument developed to assess the clinical benefit of PD treatment from the patient perspective. Specifically, it targets concepts that give meaning to PD motor symptoms in terms of their impact on everyday life. Details of its development and content validation have been published previously (4). The PD-AID was created to overcome the deficits of pre-existing instruments and developed in accordance with the United States (US) Food and Drug Administration's PRO guidance (5) with direct input from individuals with PD. The PD-AID focuses on those impacts that are direct and proximal consequences to day-to-day functioning resulting from PD motor fluctuation and areas of unmet priority related to treatment. Specifically, it assesses relevant activities of daily living (ADLs), dependence on others to perform ADLs, and life interference due to accommodating PD symptoms and treatment. The intended context of use for the PD-AID is as a clinical trial treatment benefit outcome for individuals with moderate-to-advanced PD experiencing motor fluctuations.

The PD-AID consists of an assessment to be completed in the morning and an assessment to be completed in the evening. The morning assessment comprises items addressing whether the respondent required help performing core ADLs, including getting out of bed, walking inside the home, getting on or off the toilet, showering/bathing, grooming, dressing, preparing something to eat or drink, and eating. Each of these questions is gated to determine whether the individual was able to perform the activity on their own. If no help was needed, they are instructed to indicate the level of difficulty on a 5-point categorical response scale (CRS) ranging from “not at all difficult” to “extremely difficult.” If the activity was not performed that morning, the respondent is directed to indicate if the reason was “due to Parkinson's disease” or “other reason.” Additional items focus on whether their PD caused a delay in morning activities (Yes/No) and the degree to which PD influenced the respondents' level of dependence on others or interfered with getting ready for the day (using a 7-point CRS ranging from “not at all” to “completely interfered/dependent”). The morning assessment takes ~3 min to complete. Respondents are asked to complete the morning PD-AID after they have finished their morning routine to get ready for the day, but before lunchtime.

The evening PD-AID consists of items addressing several core ADLs also included in the morning PD-AID, ADLs not previously assessed (getting in or out of a vehicle, using an electronic touchscreen), and exploratory items. It takes about 5 min to complete and uses a recall of either “since completing your morning diary” (for the items assessed in the morning) or “in the past 24 hours” (for the remaining items). As in the morning PD-AID, the items assessing ADLs are gated and use the same 5-point CRS. If the activity was not performed since completing the morning assessment or in the past 24 hours, the respondent is directed to indicate whether the reason is “due to Parkinson's disease” or “other reason.” The exploratory items address PD interference with work (if employed) and leisure activities, as well as the need to plan daily activities around expectations related to PD treatment wearing off, e.g., prevent, delay, or cease activities. Respondents are instructed to complete the evening PD-AID at night before going to bed, ideally at the same time. For both the morning and evening assessments, if a particular activity is done more than once during the given recall period, respondents are instructed to answer based on when it was at its worst or based on the instance when it was most difficult for them.

The content validity of the PD-AID as an instrument for assessing treatment benefit to important consequences of PD, i.e., day-to-day functioning and areas of unmet priority for individuals with moderate to advanced PD experiencing motor fluctuations, has been established (4). An important next step is to establish the instrument as fit-for-purpose by examining its measurement properties and determining the structure, including any subscales, and a scoring algorithm along with guidance for interpreting and defining clinically meaningful changes in scores. We report here the results of a study that explored the PD-AID's structure and scoring, internal consistency, test-retest reliability (TRTR), as well as its convergent, discriminant, and known groups validity.

## Methods

The current study was conducted as part of a 28-day, prospective non-interventional observational study. The primary objective was to gather fit-for-purpose evidence, i.e., cross sectional item- and scale-level measurement properties, for the use of the PD-AID in defining endpoints to support labeling claims. The study was conducted in accordance with ethical principles outlined in the Declaration of Helsinki. Research practices were guided by the Good Clinical Practice and regulatory requirements as applicable.

### Participants

Participants were required to be between the ages of 45 and 85 years (inclusive), have a physician-confirmed diagnosis of PD, self-reported Hoehn & Yahr (H&Y) (6) stage ≤ 3 as documented in medical records within the past year, and to be currently using and responding to a stable dose of L-Dopa ≥400 mg daily. This last requirement was reduced to ≥300 mg daily dosage on March 22nd, 2019, to help increase the recruitment rate. Participation also required the ability to recognize levodopa “wearing off,” English fluency, and a willingness and ability to comply with all study instructions and scheduled visits. A history of surgical intervention for PD (e.g., deep brain stimulation), the presence of cognitive impairment or a psychiatric condition judged by the recruiting physician as interfering with the ability to complete questionnaires for 1 month, and current or planned (within the next 2 months) participation in an interventional PD clinical trial were cause for exclusion. Efforts were made to recruit participants from 6 US-based sites (Santa Clarita, California; Boulder, Colorado; Tampa, Florida; Chesterfield, Missouri; Amherst, New York; Kirkland, Washington) who were demographically representative of a PD population with moderate-to-advanced motor fluctuation. This was accomplished by focusing recruitment efforts on obtaining an equal proportion of participants at H&Y stages 1, 2, and 3, as well as at least 20% with the presence of dyskinesia.

### Assessments

During the baseline site visit (Day 1), participants were provided with training on study procedures, PD AID completion and use of an electronic device containing the assessments to be used during the study (Table 1). Validation of the PD-AID employed both classical and modern psychometric methods. The 5-level version of the EuroQol 5-dimensional questionnaire (EQ-5D-5L) (7, 8), the patient-reported portions of the Movement Disorder Society–Unified Parkinson's Disease Rating Scale (MDS-UPDRS) (9, 10), and the Parkinson's Disease Quality of Life Questionnaire−39 (PDQ-39) (11) were used as reference measures for construct validity testing. The Patient Global Impression of Severity (PGI-S) (12) ratings at baseline served to facilitate a known-groups analysis. Data for all participants were used to estimate test-retest reliability.

**Table 1:** Schedule of assessments.

**Instrument**	**Recall period**	Day 1^*^	**Day 14**	**Day 28**	**Final visit^†^**
Location		At site	At home	At home	At site
EQ-5D-5L	Today	✓	——–	✓	Return device
MDS-UPDRS^‡^	1 week	✓	✓	✓	Return device
PD-AID	This morning for the Morning PD-AID Since completing the Morning PD-AID or Past 24 hours for Evening PD-AID	✓Daily morning and evening			
PDQ-39	Prior month	✓	——–	✓	✓
PGI-S		✓	✓	——–	✓

* *Baseline/consent visit.*

† *Final visit = Day 29 or up to 4 days after Day 28.*

‡ *Patient-reported portions (Parts I & II).*

*EQ-5D-5L, 5-level version of the EuroQol 5-dimensional questionnaire; MDS-UPDRS, Movement Disorder Society–Unified Parkinson's Disease Rating Patient Reported Outcomes Portion; PD-AID, Parkinson's Disease Activities, Interference, and Dependence Instrument; PDQ-39, Parkinson's Disease Quality of Life Questionnaire; PGI-S, Patient Global Impression of Severity*.

### Psychometric Analyses

Unless otherwise specified, all analyses employed R version 3.6.1 or higher (13). Item descriptive statistics were generated using a combination of base features from R and the R package “psych” (14). Psychometric models were fit using the R package “mirt” version 1.30 or higher (15).

#### Item-Level Analyses

##### Preliminary Descriptive and Diagnostic Evaluations

Descriptive analyses of the items comprising the PD-AID focused on examining the overall response distribution for each item, floor and ceiling effects, and missingness on Day 1. The ADL questions in the PD-AID include branching logic, meaning that a response to the lead question for a concept determines subsequent queries related to that concept. Thus, while multiple questions appear, together they address a single concept. Branching logic was used to reduce respondent burden when completing the instrument and to avoid illogical data. For purposes of examining missingness and response distributions, the responses to the multiple questions related to the PD-AID ADL concepts were collapsed into a 7-point scale representing the full range of possible responses (Supplementary Figure 1). Although this type of question can lessen participants' response burden, it can also result in sparseness. Therefore, item responses were coded across the 7-point scale such that an ordinal variable representing a concept was analyzed.

Across all of the PD-AID items, floor, and ceiling effects were established on an item-by-item basis, based on whether the lowest or highest response category was endorsed by more than $\frac{1}{k}\times 100\%$ of respondents where *k* represented the number of possible response categories for an item. Inter-item polychoric correlations were estimated to assess whether responses to the items conformed to *a priori* expectations and to help identify items that might behave erratically.

#### Structure and Scoring

The dimensionality of the PD-AID was examined at Day 1 using full-information exploratory item factor analysis (16). The morning and evening assessments were factored separately and unidimensional confirmatory models were supported by model fit statistics and the Empirical Kaiser Criterion (17).

To establish scoring that would represent a meaningful outcome for patients and be useful to clinicians and researchers, 4 scores, supported by the original qualitative development research (4), were computed. The first score was comprised of the 8 morning ADL items (AM8). The second set of scores contained the AM8 items plus 2 additional morning items related to interference due to PD and overall dependence on others (AM10). The third set of scores contained the 6 evening ADL items (PM6). The final score was an average of the 4 ADL items common to the morning and evening PD-AID for an ADL total score (ADL8) (Table 2). Each of the scores were calculated as unweighted classical test theory (CTT) scores, using the sums of coded item responses for the AM8, AM10, and PM6, and the mean of the sums of the 4 in-common ADL item responses for the ADL8 score.

**Table 2:** Potential PD-AID scores.

**Item concepts**	**Candidate score**			
	**AM8**	**AM10**	**PM6**	**ADL8**
Getting out of bed	X	X		
Walking inside home	X	X	X	X
Getting on/off toilet	X	X	X	X
Showering and bathing	X	X		
Grooming	X	X		
Dressing	X	X		
Preparing food	X	X	X	X
Feeding oneself	X	X	X	X
Getting in/out of vehicle			X	
Using electronic device			X	
PD interference		X		
Overall dependence		X		

*ADL, Activities of Daily Living; AM, morning; PD, Parkinson's disease; PD-AID, Parkinson's Disease Activities of Daily Living, Interference and Dependence;. PM, evening*.

#### Scale-Level Analyses

Internal consistency was estimated at Baseline. Specifically, Cronbach's coefficient α (18) was calculated for the CTT scores. A coefficient value of α ≥ 0.70 was considered acceptable. TRTR estimates were calculated using the 2-way random intraclass correlation coefficient [ICC (1, 2)] (19) between week 1 and week 4. Values of ICC (1, 2) ≥ 0.70 were considered sufficient for demonstrating TRTR validity. The choice of the ICC (1, 2) formulation was employed to accommodate variability between participants in their last analysis day (20). Convergent and discriminant validity was assessed cross-sectionally using Spearman correlations (21). Specifically, PD-AID scores were correlated with the Total and 8 subscale scores (Mobility, ADLs, Emotional well-being, Stigma, Social support, Cognition, Communication, Bodily discomfort) of the PDQ-39; scores for the 2 patient-reported subscales of the MDS-UPDRS (Motor and Non-motor Experiences of Daily Living); and scores on the 5 dimensions (Mobility, Self-care, Usual Activities, Pain/discomfort, Anxiety/depression) and the visual analog scale (VAS) of the EQ-5D-5L.

Convergent correlations were expected for PDQ-39 Total, Mobility, and ADL scores; the patient-reported Motor Experiences of Daily Living MDS-UPDRS scores; and the Self-care, Usual Activities and Mobility EQ-5D-5L index scores as these instruments include some related concepts to those covered by the PD AID.

Discriminant correlations were expected between the PD-AID scores and the PDQ-39 Emotional Well-being, Stigma, Social Support, and Bodily Discomfort; the patient-reported portion of the MDS-UPDRS Non-motor Aspects of Daily Living; and the EQ-5D-5L Depression/anxiety, Pain/discomfort subscale scores, and the VAS. We had no a priori expectations for the correlations for the remaining reference instrument subscale scores.

Known-groups validity effects were obtained by calculating summary statistics for the PD-AID scores within participants' ratings on the PGI-S at Baseline.

## Results

### Demographic and Health Information

A total of 94 participants enrolled in the non-interventional study (Table 3). Ninety-three (mean age 69 years; 68% male; 91% White) had data for at least one administration of the PD-AID. Using PD-specific data from the participants' medical records, clinicians reported 34 participants (37.6%) with dyskinesia associated with L-Dopa use. The mean time since PD diagnosis was 6.9 years (SD = 4.2; range 0.3–20 years).

**Table 3:** Participant-reported demographic and health information.

**Characteristic**	**Total sample** ***N* = 93**
Age, (years) Average (SD)	68.8 (8.6)
Gender, *n* (%)	
Male	63 (67.7%)
Female	30 (32.3%)
Race (all that apply selected), *n* (%)	
White	85 (91.4%)
Asian	4 (4.3%)
Black or African American	2 (2.2%)
American Indian or Alaska Native	1 (1.1%)
Not reported	1 (1.1%)
Ethnicity, *n* (%)	
Not Hispanic/Latino(a)	90 (96.8%)
Hispanic/Latino (a)	2 (2.2%)
Not reported	1 (1.1%)
Highest level of education, *n* (%)	
High school (no degree) or less	1 (1.1%)
High school graduate (or equivalent)	11 (11.8%)
Some college (no degree)	27 (29.0%)
Associate degree	7 (7.5%)
Bachelor's degree	28 (30.1%)
Master's degree	15 (16.1%)
Doctoral degree	4 (4.3%)
Work status (all that apply selected), *n* (%)^*^	
Retired	60 (64.5%)
Working full-time	12 (12.9%)
On disability	9 (9.7%)
Working part-time	6 (6.5%)
Part-time/Retired	2 (2.2%)
Homemaker	2 (2.2%)
Unemployed	1 (1.1%)
Other (reported as self-employed)	1 (1.1%)
General health status, *n* (%)	
Excellent	11 (11.8%)
Very good	25 (26.9%)
Good	47 (50.5%)
Fair	8 (8.6%)
Poor	2 (2.2%)
Means of assistance (if any), *n* (%)	
Spouse/partner	45 (48.4%)
Other family members	5 (5.4%)
Paid help	4 (4.3%)
Friends	2 (2.2%)
Spouse/Family/Friend/Paid	1 (1.1%)
Volunteer help	1 (1.1%)
Other (not specified)	34 (36.6%)
Not reported	1 (1.1%)
Hoehn and Yahr stage, *n* (%)	
Stage 1 (Unilateral symptoms only)	31 (33.3%)
Stage 2 (Bilateral symptoms; no balance or walking problems)	20 (21.5%)
Stage 3 (Problems with balance and walking)	37 (45.2%)

* *Work status sum is >100.0% as one participant selected both “working part-time” and “retired.”*

One participant did not provide any PRO data, and therefore, was not included in the analyses; this participant reported that the administration schedule for the assessments was burdensome. Six participants who had data for at least one administration of the PD-AID dropped out of the study for the following reasons: burdensome administration schedule (*n* = 4); difficulty entering answers into electronic device due to PD symptoms (*n* = 1); the electronic device was too difficult to understand how to use (*n* = 1). While these 6 participants dropped out before their last study visit, their available data from Baseline visit until dropout date were used in subsequent analyses.

### Psychometric Analyses

The results of the psychometric analyses are shown in Table 4.

**Table 4:** Psychometric analyses.

**Analysis**	**• Procedures** **• Prespecified acceptability criterion**	**Results**
Descriptive statistics	• Frequencies and other summaries • None	Skewed responses and sparseness noted
Structure and scoring	• Comparison to conceptual framework with inter-item correlations and confirmatory full-information factor model with single factors at first assessment • Acceptable fit statistics and interpretable structure	Scoring determined by sums • AM8: 8 ADL items • AM10: 10 items • PM6: 6 ADL items • ADL8: 4 ADL items common to AM and PM PD-AID
Internal consistency	• Interpretation of reliability statistic (Cronbach's alpha [α]) • α>0.7	• AM8: α = 0.93 • AM10: α = 0.94 • PM6: α = 0.73 • ADL8: α = 0.86
Test-Retest reliability	• Shrout and Fleiss (19); *ICC*(2, 1) between Weeks 1 and 4 • *ICC*(2, 1)≥ 0.7	• AM8: *ICC*(2, 1) = 0.56;95*% CI* = (0.47, 0.69) • AM10: *ICC*(2, 1) = 0.59;95*% CI* = (0.55, 0.75) • PM6: *ICC*(2, 1) = 0.66;95*% CI* = (0.43, 0.66) • ADL8: *ICC*(2, 1) = 0.52;95*% CI* = (0.39, 0.64)
Convergent validity	• Spearman correlation between scores at Day 1 and Last Visit for PD-AID, PDQ-39, and EQ-5D-5L • Correlations in the expected direction with co-validating measures • Correlations stronger between scores from more theoretically overlapping constructs • At least an |r| ≥ 0.40	*Convergent validity* Spearman correlations between the 4 candidate PD-AID scores and the following were (0.41 ≤ |r| ≤ 0.83) PDQ-39 • Total (0.65 ≥ |r| ≥ 0.53) • Mobility (0.83 ≥ |r| ≥ 0.61) • ADLs (0.72 ≥ |r| ≥ 0.63) MDS-UPRS • Motor experiences of daily living (0.70 ≥ |r| ≥ 0.63) • Non-Motor experiences of daily living (0.48 ≥ |r| ≥ 0.44) EQ-5D-5L • Mobility (0.72 ≥ |r| ≥ 0.61) • Self-care (0.63 ≥ |r| ≥ 0.55) • Usual activities (0.69 ≥ |r| ≥ 0.57) • Pain/discomfort (0.54 ≥ |r| ≥ 0.41) • Anxiety/Depression (0.50 ≥ |r| ≥ 0.35) • VAS (0.55 ≥ |r| ≥ 0.41)
Discriminant validity	• Spearman correlation between scores at Day 1 and Last Visit for PD-AID, PDQ-39, and EQ-5D-5L • |*r*|≥0.4 (evaluated by pairwise comparisons)	Spearman correlations between the 4 Candidate PD-AID scores and the following PDQ-39 subscale scores were (0.20 ≤ |r| ≤ 0.38). • Emotional Well-being (28 ≤ |r| ≤ 0.38) • Stigma (0.20 ≤ |r| ≤ 0.30) • Social Support (0.23 ≤ |r| ≤ 0.35) • Bodily Discomfort |r| = 0.29
Known-Groups validity	• Descriptively comparing Baseline PD-AID scores across PGI-S levels • Monotonic ordering in the expected direction	AM8, AM10, PM6, and ADL8 ordered monotonically across Baseline PGI-S

*Correlation Matrices are available as Supplemental Material.*

*ADL, Activities of Daily Living; AM, morning; EQ-5D-5L, 5-level version of the EuroQol 5-dimensional questionnaire; MDS-UPDRS, Movement Disorder Society–Unified Parkinson's Disease Rating Patient Reported Outcomes Portion; PD, Parkinson's disease; PD-AID, Parkinson's Disease Activities of Daily Living, Interference and Dependence; PD-AID, Parkinson's Disease Activities, Interference, and Dependence Instrument; PDQ-39, Parkinson's Disease Quality of Life Questionnaire; PGI-S, Patient Global Impression of Severity; PM, evening*.

#### Item-Level Analyses

Overall, completion of the morning PD-AID was higher than the evening PD-AID. The morning PD-AID completion dropped off from 93/93 on the first analysis day to 86/93 on the last analysis day, while the evening PD-AID showed an increase in completion (from 65 to 76) across those same days. Last study day was defined as a participant's last PD-AID administration within the window of days 28–33.

Item-wise completion rates were generally high across all items, with the exception of the item “require help to bathe” from the morning administration, and the item “require help to prepare food” from the evening administration. Irrespective of time of analysis, both the morning and evening administrations showed floor effects across all items and extreme sparseness in the higher response options.

Inter-item polychoric correlations were examined for the PD-AID morning and evening items. All morning items were positively correlated with one another (Supplementary Figure 2A). One item from the morning assessment (delay activities because of PD), employed dichotomous response options, whereas all other items had a potential of 5-categorical responses. As such, this item was excluded from the correlation analysis.

The PD-AID evening items showed a markedly different pattern of associations than the morning items (Supplementary Figure 2B). First, most, but not all, items showed positive associations with one another. Second, sporadic negative correlations were observed throughout the correlation matrix, with one item (confidence that medication would work) showing unexpected negative correlations with all but 3 of the other items. Third, the magnitude of the correlations observed in the evening items was less than the morning items, with working for pay and confidence that medication would work showing the starkest deviations from the remaining items. After reviewing these results and the content validity of the PD-AID items, it was determined that for the evening administrations, only the ADL items would be used for any modeling and scoring going forward (the non-ADL items would still be included in the PD-AID, but only for descriptive purposes). Therefore, 6 evening items were retained in further analyses.

#### Structure and Scoring

Based on team discussions and the item response theory (IRT) modeling results, it was decided that CTT sum scores would be generated for the PD-AID items. Unidimensional models were the basis for 4 sum (CTT) scores of available items (Table 2). These CTT scores (i.e., AM8, AM10, PM6, and ADL8) were supported by the IRT results because factor loadings across items within models were similar in magnitude.

#### Scale-Level Analyses

Descriptive statistics for the PD-AID scores at Baseline and the last analysis day (day 28–33) are available in Supplementary Table 1. Score values differ as the number of items potentially contributing to the sums differ. In all cases, values showed a right skew with means and medians closer to the minimum value of 0, which was a result of the floor effects observed across the individual items.

Correlations were calculated to show the relationship between the 4 possible scoring options. The magnitude of the correlation between the scores was moderate to strong, with the lowest pairwise value of *r* = 0.69 between AM8 and PM6 and the highest pairwise value of *r* = 0.97 between AM10 and AM8. Notably, the associations between daily ADL scores were consistently high across all scores (*r* ≥ 0.86).

Internal consistency for the measure was assessed at Day 1 using Coefficient α, which implies equal weighting of the items in the CTT scoring regimes. All scores had internal consistency estimates that exceeded the criteria α ≥ 0.70 (Table 4). Test-retest indices were estimated with the ICC (1, 2) using scores for Day 1 and the last day analysis. All participants were used in the calculation of TRTR. All ICC (1, 2) estimates were variable, with the point estimates ≤ 0.70. While the 95% confidence interval for the AM8 covered the threshold criterion for acceptable TRTR and the correlation levels represent a large effect size, more values within the 95% confidence interval were below the threshold of acceptability (22).

Correlations between the 4 candidate PD-AID scores and the PDQ-39 Total, Mobility, and ADLs, the indexes of the EQ-5D-5L—with one exception, and the 2 patient-reported MDS-UPDRS subscores were indicative of convergent validity (0.41 ≤ |*r*| ≤ 0.83). The single exception for these measures was the correlation between the PM6 and the EQ-5D-5L anxiety and depression score (*r* = 0.35). Discriminant validity was indicated by the correlations between the PD-AID scores and the PDQ-39 Emotional Well-being, Stigma, Social Support, and Bodily Discomfort subscales (0.20 ≤ |*r*| ≤ 0.38).

Known groups validity was assessed by calculating the summary statistics for the PD-AID scores within strata created by participants' responses to the Day 1 PGI-S. The results aligned with expectations, as the average for all PD-AID scores increased monotonically across the PGI-S ratings for PGI-S groups that had multiple response (i.e., PGI-S = 4 only had one respondent for each of the 4 PD-AID scores). Results indicate the all-candidate PD-AID scores exhibited known groups validity (Supplementary Table 2).

## Discussion

This study was conducted to explore and evaluate the measurement performance of the patient-reported PD-AID Instrument, the content of which has been developed for use as a measure of clinical benefit in clinical trials of individuals with PD who are experiencing motor fluctuations. This work assessing the psychometric performance of potential PD-AID scores adds to the fit for purpose evidence of previous work which documented the qualitative content validity of the PD-AID.

Completion rates of the PD-AID were generally high across all items, with the exception of the item “require help to bathe” from the morning administration, and the item “require help to prepare food” from the evening administration. In retrospect, the completion rate of the bathing item may be showing a higher missingness rate because daily bathing might be as much a personal preference as it is impacted by PD. Similarly, the “help to prepare food” item from the evening PD-AID missingness may reflect changes in eating patterns and preferences not necessarily related to PD, e.g., eating meals earlier in the day or having pre-prepared meals stored in the refrigerator or freezer.

Structural analyses supported the 4 possible scores defined in Table 2 and a simple sum of available items scoring method. All 4 scoring algorithms exhibited psychometric properties that were indicative of acceptable performance. However, the AM10 showed marginally better score quality in the form of higher correlations in the analysis of concurrent validity and higher estimated reliability.

Internal consistency analyses for all 4 scores showed strong results indicating the items were consistently measuring a unidimensional construct. Test-retest reliability estimates, using a 4-week time-lag, were weaker than expected, perhaps due to the long inter-visit period, thus allowing some additional measurement error in the data. However, the values were not significantly lower than the usual criterion for stable measurements over time. Also, there was attrition across the study that could have attenuated these results.

Correlations between the PD-AID and other validated measures were in the expected directions and showed moderate to strong values, with PD-AID scores showing highest correlation with constructs measuring concepts such as activities and mobility—indicating that the PD-AID scores are themselves measures of ADL. PD-AID scores also showed the expected pattern when stratified by Baseline PGI-S ratings in that mean scores increased as severity ratings increased, indicating that the scores were able to distinguish patient-perceived severity in this patient set.

The main limitation to this study is that the participants had less advanced PD than originally planned. This was likely due to the fact that patients with severe PD symptoms were less willing to participate because of the study length and burden of answering questions electronically on a daily basis. Additionally, the sample size was smaller than planned, which could also have contributed adversely to psychometric analyses. The non-interventional nature of the study precluded an evaluation of responsiveness to intervention. We recommend further research to replicate these results and to establish outstanding measurement properties and interpretation thresholds for meaningful changes in PD-AID scores. While our conclusion is that the proposed PD-AID scoring based upon this data is psychometrically sound, we recommend further validation efforts to support its use in persons with more advanced PD. In addition, exploring the PD AID measurement performance in a more racially and ethnically diverse sample is recommended. Finally, we do not consider the current study to constitute the definitive validation of the PD-AID. We expected our ICC (1, 2) for evaluating TRTR to be > 0.7 and we also expected to have less of a floor effect on the items than was observed. This observed range restriction could be a reason for an observed ICC (1, 2) to be attenuated. Further, although future studies may lead to improvements to the scoring, based on this research, we constructed multiple scores allowing for flexibility of use for researchers and clinicians using the instrument. We recommend further evaluation of TRTR in a sample of participants exhibiting wider variability in scores to improve evaluation of TRTR performance.

Missing data is always a concern in clinical research studies and although compliance rates were ~70% or higher missing responses could have affected the results. Some patients who discontinued their participation in the study early, cited frequency of the administration schedule as a reason for exiting the study (*n* = 5) and therefore some consideration should be taken in future studies to ensure the schedule is sufficient to address the research hypothesis and flexible with regards to considerations of patient burden. We also strongly recommend that future psychometric research for the PD-AID be in larger sample sizes. For future use, should compliance rates for completing the PD-AID be below 70%, the sum scoring method may negatively affect how well the PD-AID scores are measuring the constructs of interest. Further, because the study sample was required through eligibility criteria to be US English speaking, cultural and linguistic differences in the responses to the PD-AID should be further investigated using rigorous psychometric methods (i.e., tests for differential item functioning) in the expanded samples.

In conclusion, the cross-sectional measurement properties for the PD AID show promise with regards to internal consistency, construct, and discriminate validity. This conclusion should be considered in light of the discussed limitations. Additional evaluation of the scale structure using the full longitudinal data and employing modern test theory methods are expected to yield greater support for the scoring and TRTR.

## Data Availability Statement

The raw data supporting the conclusions of this article will be made available by the authors, without undue reservation.

## Ethics Statement

The studies involving human participants were reviewed and approved by the protocol was submitted to appropriate local Institutional Review Boards (IRBs; Copernicus Group for 5 sites; University of Florida for 1 site) for ethics review and approval prior to any contact with participants. The patients/participants provided their written informed consent to participate in this study.

## Author Contributions

LD: research project: conception, organization, execution; statistical analysis: design, execution, review and critique; and manuscript preparation: writing of the first draft, review and critique. DA: research project: organization; statistical analysis: design, execution, review and critique; and manuscript preparation: review and critique. DM: research project: conception, organization, execution; statistical analysis: review and critique; and manuscript preparation: review and critique. NJ and CE: research project: organization, execution; statistical analysis: review and critique; and manuscript preparation: review and critique. BF: statistical analysis: design, execution, review and critique; and manuscript preparation: review and critique. All authors contributed to the article and approved the submitted version.

## Funding

This study was funded by Pfizer Inc. Writing and editorial assistance was provided by Maria B. Vinall of Medical Communications Depot, Inc. Ms Vinall's services were paid for by Pfizer.

## Conflict of Interest

LD and DM are currently employees of Pfizer Inc. and were so employed at the time the study was conducted. Their compensation includes company stock. DA, CE, BF, and NJ are, and were at the time of study, employees of Endpoint Outcomes, which conducted the study. The authors declare that this study received funding from Pfizer Inc. The funder had the following involvement in the study: Authors LD and DM were employees of the funder. To the extent that these co authors represent the funder, the funder involvement is listed in the author contributions statements.

## Publisher's Note

All claims expressed in this article are solely those of the authors and do not necessarily represent those of their affiliated organizations, or those of the publisher, the editors and the reviewers. Any product that may be evaluated in this article, or claim that may be made by its manufacturer, is not guaranteed or endorsed by the publisher.
