# Supplementary material for: The Measurement Performance of the Parkinson's Disease Activities of Daily Living, Interference, and Dependence Instrument
Source: Front Neurol. 2022 Mar 31;13:760174. doi: 10.3389/fneur.2022.760174 (PMC9009412; doi:10.3389/fneur.2022.760174)
Supplement: Supplementary file 1 [file Table_1.DOCX]

**Supplemental Table 1 Descriptive Statistics for the PD-AID Scores**

| **When Assessed** | **Score** | **Range*** | **n** | **Mean** | **SD** | **Min** | **Median** | **Max** |
| --- | --- | --- | --- | --- | --- | --- | --- | --- |
| **Day 1** | AM8 | 0–48 | 98 | 8.0 | 10.29 | 0 | 4.5 | 41 |
|  | AM10 | 0–60 | 98 | 10.3 | 12.59 | 0 | 7.4 | 51 |
|  | PM6 | 0–36 | 65 | 5.0 | 5.23 | 0 | 5.9 | 20 |
|  | ADL8 | 0–48 | 98 | 3.4 | 4.83 | 0 | 2.6 | 20 |
| **Last analysis**  **(Day 28-33)** | AM8 | 0–48 | 82 | 5.3 | 6.71 | 0 | 4.5 | 30 |
|  | AM10 | 0–60 | 82 | 7.2 | 8.59 | 0 | 5.9 | 38 |
|  | PM6 | 0–36 | 39 | 4.6 | 5.75 | 0 | 4.5 | 22 |
|  | ADL8 | 0–48 | 86 | 2.6 | 3.38 | 0 | 1.5 | 14 |

ADL, activities of daily living; AM, morning; Max, maximum; Min, minimum; PD-AID, Parkinson’s Disease Activities of Daily Living, Interference and Dependence; PM, evening

*Scores were based on the original response options
