# Supplementary material for: The Measurement Performance of the Parkinson's Disease Activities of Daily Living, Interference, and Dependence Instrument
Source: Front Neurol. 2022 Mar 31;13:760174. doi: 10.3389/fneur.2022.760174 (PMC9009412; doi:10.3389/fneur.2022.760174)
Supplement: Supplementary file 2 [file Table_2.DOCX]

**Supplemental Table 2 Known Groups Assessment for the PD-AID at Baseline**

| Score | Range | PGI-S | n | Mean | SD | Minimum | Median | Maximum |
| --- | --- | --- | --- | --- | --- | --- | --- | --- |
| AM8 | 0-48 | 0 | 19 | 0.68 | 1.376 | 0 | 0.0 | 4 |
|  |  | 1 | 36 | 5.33 | 7.910 | 0 | 2.5 | 40 |
|  |  | 2 | 26 | 10.12 | 9.721 | 0 | 7.5 | 40 |
|  |  | 3 | 11 | 14.27 | 8.014 | 0 | 18.0 | 25 |
|  |  | 4 | 1 | 2.00 | NA | 2 | 2.0 | 2 |
| AM10 | 0-60 | 0 | 19 | 0.89 | 1.696 | 0 | 0.0 | 6 |
|  |  | 1 | 36 | 6.83 | 9.419 | 0 | 3.5 | 47 |
|  |  | 2 | 26 | 12.69 | 11.770 | 0 | 9.5 | 48 |
|  |  | 3 | 11 | 19.00 | 9.381 | 3 | 21.0 | 30 |
|  |  | 4 | 1 | 7.00 | NA | 7 | 7.0 | 7 |
| PM6 | 0-36 | 0 | 19 | 0.69 | 1.580 | 0 | 0.0 | 5 |
|  |  | 1 | 36 | 3.38 | 3.853 | 0 | 2.0 | 13 |
|  |  | 2 | 26 | 7.06 | 4.973 | 0 | 7.0 | 17 |
|  |  | 3 | 11 | 10.55 | 5.007 | 2 | 11.0 | 20 |
|  |  | 4 | 1 | 11.00 | NA | 11 | 11.0 | 11 |
| ADL8 | 0-48 | 0 | 19 | 0.18 | 0.448 | 0 | 0.0 | 1.5 |
|  |  | 1 | 36 | 2.01 | 3.739 | 0 | 1.0 | 20 |
|  |  | 2 | 26 | 4.31 | 4.313 | 0 | 2.5 | 20 |
|  |  | 3 | 11 | 6.45 | 3.029 | 2 | 7.0 | 10 |
|  |  | 4 | 1 | 4.50 | NA | 4.5 | 4.5 | 4.5 |

ADL, activities of daily living; AM, morning; Max, maximum; Min, minimum; PD-AID, Parkinson’s Disease Activities of Daily Living, Interference and Dependence; PGI-S, Patient Global Impression of Severity; PM, evening; SD, standard deviation
