# Supplementary figures and images for: The Measurement Performance of the Parkinson's Disease Activities of Daily Living, Interference, and Dependence Instrument
Source: Front Neurol. 2022 Mar 31;13:760174. doi: 10.3389/fneur.2022.760174 (PMC9009412; doi:10.3389/fneur.2022.760174)

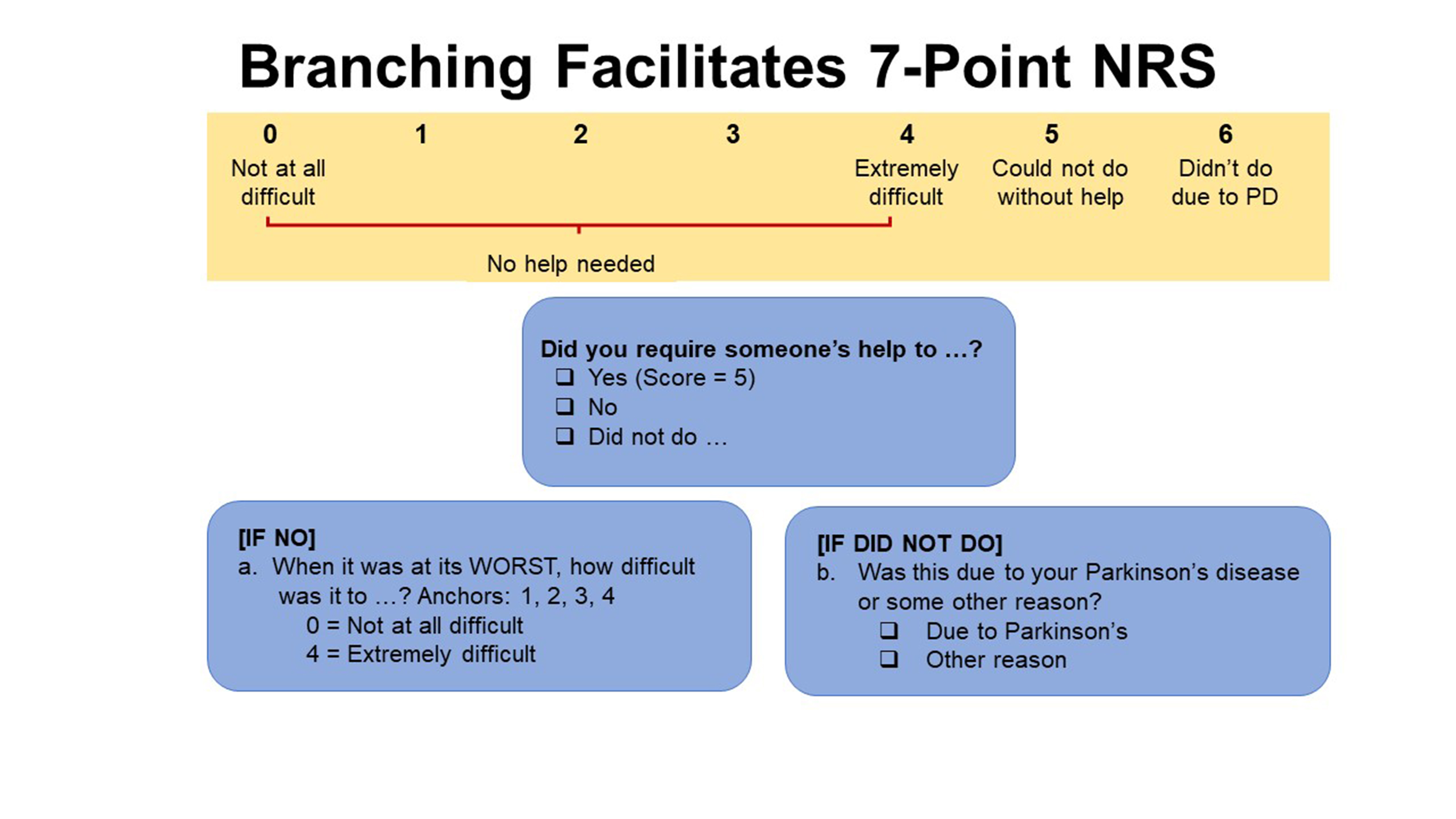

Supplement: Supplementary file 3 [file Image_1.JPEG]

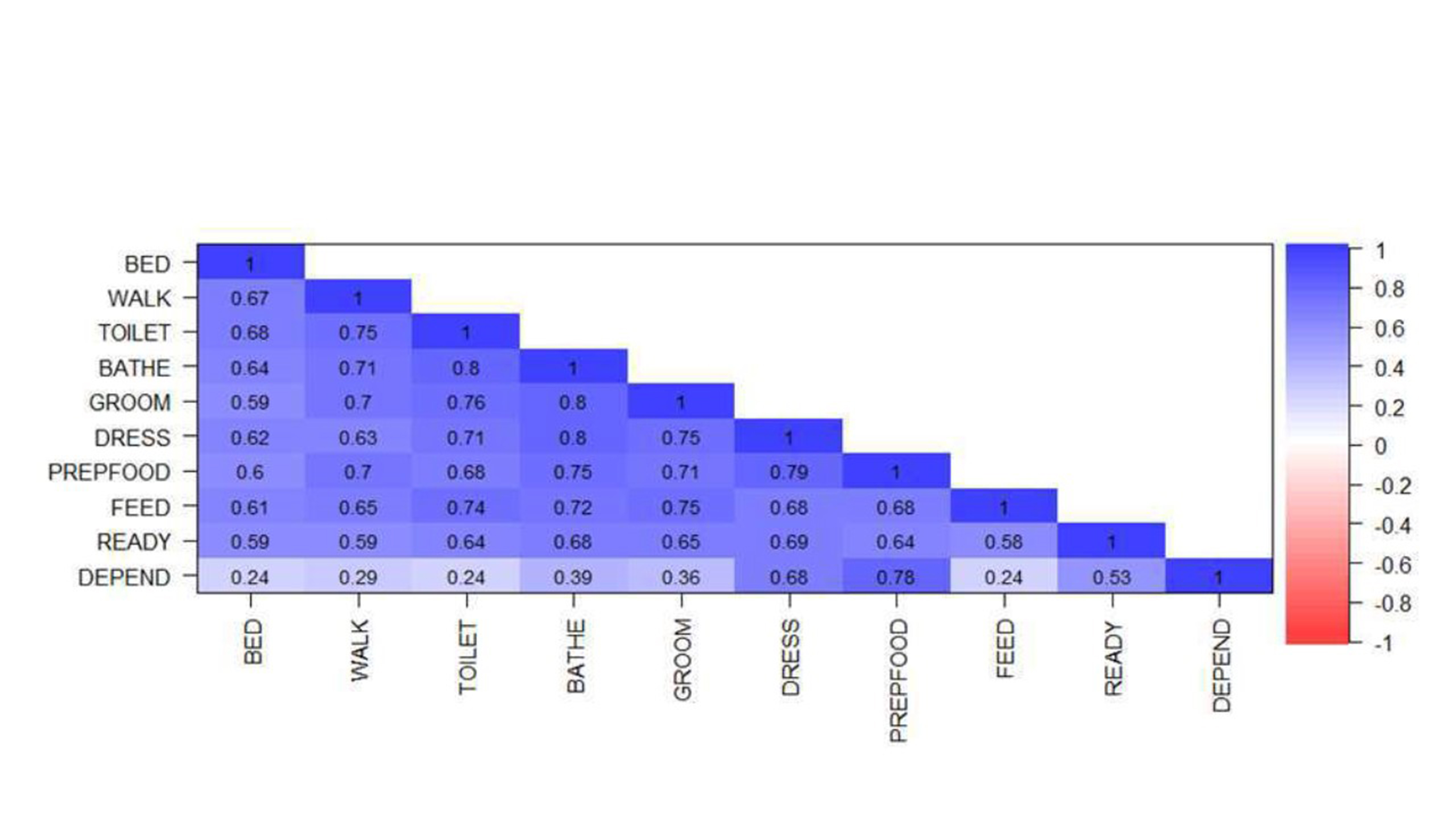

Supplement: Supplementary file 4 [file Image_2.JPEG]

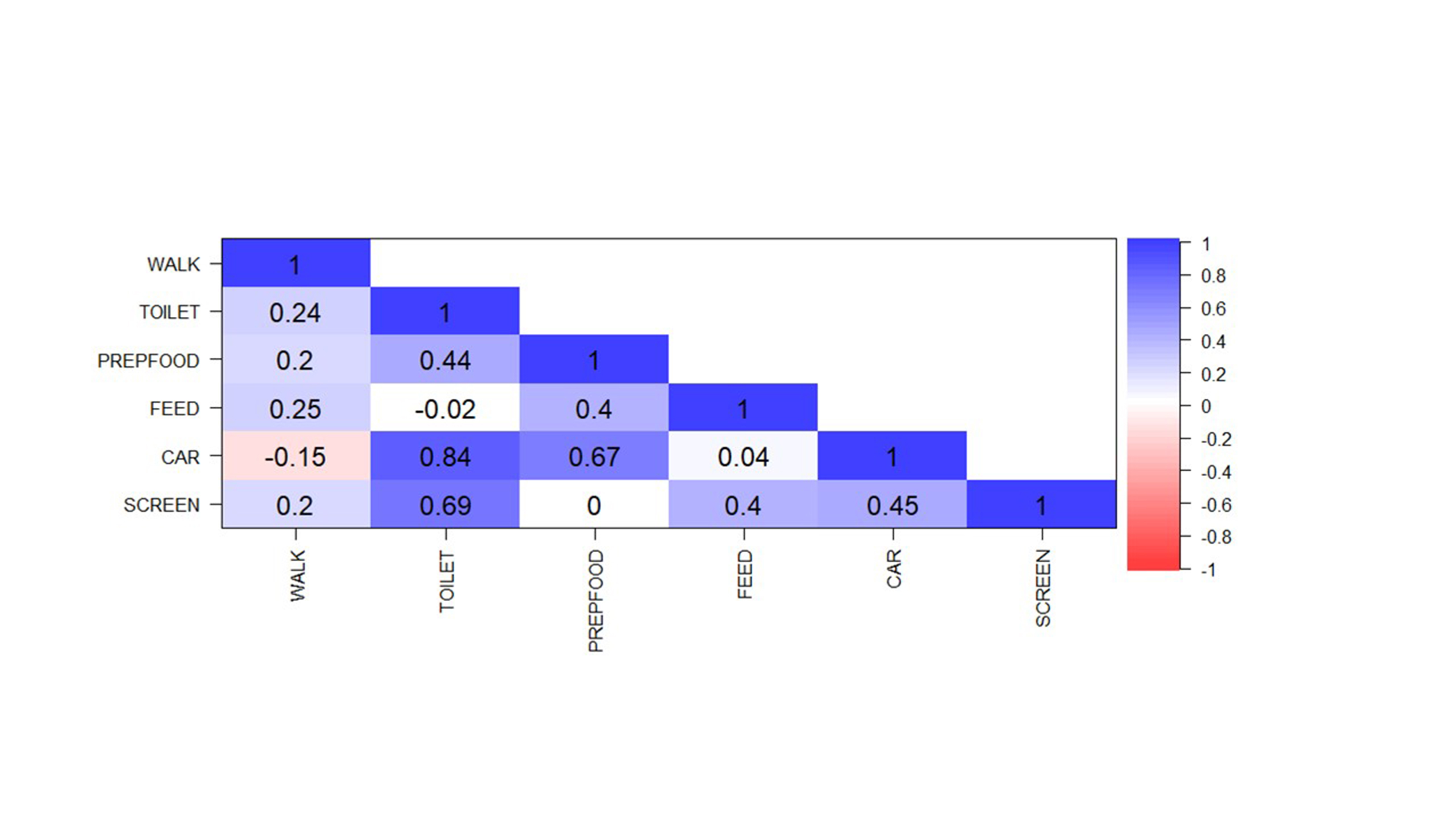

Supplement: Supplementary file 5 [file Image_3.JPEG]
